# Supplementary material for: Anti-TNF Therapies Suppress Adipose Tissue Inflammation in Crohn’s Disease
Source: Int J Mol Sci. 2022 Sep 22;23(19):11170. doi: 10.3390/ijms231911170 (PMC9570367; doi:10.3390/ijms231911170)
Supplement: Supplementary file 1 [file ijms-23-11170-s001.zip › Supplementary Table S1.pdf]

Supplementary Table S1. Immunophenotypic profile of human adipose-derived stem cells (ASCs) isolated from CD subjects treated or untreated with anti-TNF drugs. The values reflect the mean ±SD percentage surface positive staining of ASCs for a part of surface antigens including hematopoietic and stromal markers.

Antibody	Without anti-TNF CD-ASCs	Anti-TNF treated CD-ASCs
**CD34**	0,1 ± 0,07	0,1 ± 0,09
**CD45**	0,1 ± 0,06	0,1 ± 0,08
**CD73**	97,6 ± 0,4	96,9 ± 0,4
**CD90**	97,1 ± 0,8	97,5 ± 0,7
**CD105**	98,9 ± 0,9	98,3 ± 0,4
