# Supplementary material for: Anti-TNF Therapies Suppress Adipose Tissue Inflammation in Crohn’s Disease
Source: Int J Mol Sci. 2022 Sep 22;23(19):11170. doi: 10.3390/ijms231911170 (PMC9570367; doi:10.3390/ijms231911170)
Supplement: Supplementary file 1 [file ijms-23-11170-s001.zip › Supplementary Table S2.pdf]

Supplementary Table S2. Human gene expression analysis. Results were calculated using the comparative Ct method and expressed relative to the expression of the housekeeping gene 18S (Hs03928985_g1).

Detector	Gene name
Inflammatory markers	
TNF-A-Hs01113624_g1	*Tumor necrosis factor alpha*
MCP-1/CCL2-Hs00234140_m1	*Monocyte chemotactic protein 1*
IL-6-Hs00985639_m1	*Interleukin-6*
IL-1B-Hs00174097_m1	*Interleukin-1 beta*
IL-17-Hs00174383_m1	*Interleukin-17*
IL-23-Hs00372324_m1	*Interleukin-23*
IL-33-Hs04931857_m1	*Interleukin-33*
IL-34-Hs01050926_m1	*Interleukin-34*
IL-12b-Hs01011518_m1	*Interleukin-12b*
Anti-inflammatory markers	
IL-10-Hs00961622_m1	*Interleukin-10*
ADIPOQ-Hs00605917_m1	*Adiponectin*
G-CSF-Hs00738432_m1	*Granulocyte-colony stimulating factor*
TGFB1-Hs 00998133_m1	*Transforming growth factor beta 1*
Invasion markers	
MMP2-Hs01548727_m1	*Metalloproteinase-2*
MMP9-Hs00234579_m1	*Metalloproteinase-9*
Antigen Presentacion markers	
CIITA-Hs00172094_m1	*Class II Major Histocompatibility Complex Transactivator*
HLA-DM-Hs00157943_m1	*Class II Major Histocompatibility Complex, DM alpha*
HLA-DR-Hs04192464_m1	*Class II Major Histocompatibility Complex, DR alpha*
HLA-DPB-Hs03045105_m1	*Class II Major Histocompatibility Complex, DP beta 1*
CD-74-Hs00269961_m1	*HLA class II Histocompatibility antigen gamma chain*
CD-80-Hs01045161_m1	*T-lymphocyte activation antigen CD80*
CD-40-Hs01002915_g1	*CD40 ligand*
